# Caregivers’ early stimulation behaviors on early child development outcomes in Northern Ghana

**DOI:** 10.1371/journal.pone.0344687

**Published:** 2026-04-03

**Authors:** Eunsoo Timothy Kim, John A. Gallis, Mohammed Ali, Margaret Lillie, Safiyatu Abubakr-Bibilazu, Haliq Adam, Elena McEwan, John Koku Awoonor-Williams, John Hembling, Joy Noel Baumgartner

**Affiliations:** 1 ChildFund International, Richmond, Virginia, United States of America; 2 Duke University, Durham, North Carolina, United States of America; 3 College of Community and Organization Development, Walewale Campus, Ghana; 4 University of North Carolina at Chapel Hill, Chapel Hill, North Carolina, United States of America; 5 Chemonics International Inc., Accra, Ghana; 6 Catholic Relief Services Country Office, Tamale, Ghana; 7 Catholic Relief Services Headquarters, Baltimore, Maryland, United States of America; 8 Ghana College of Physicians and Surgeons, Accra, Ghana; Jawaharlal Institute of Postgraduate Medical Education and Research, INDIA

## Abstract

Despite the government prioritizing Early Childhood Development (ECD) in Ghana, data on responsive caregiving is limited. This study examined the type and extent of Early Stimulation Activities (ESAs) practiced by mothers, and other caregivers and the associations between ESAs and children’s development by caregiver type in Northern Ghana. Data were from a cluster-randomized controlled trial with mother-child dyads that evaluated the impact of an integrated maternal mental health and early childhood intervention program. The primary outcome was measured by the Developmental Milestone Checklist (DMC-II). Covariates included maternal report of ESAs performed by herself, child’s father, and other caregivers. Linear regression analyses with cluster-adjusted standard errors were performed. Nearly 15% of mother-participants (n = 313) reported 4 + ESAs in the past 3 days. Within the last 3 days, on average, women reported 2.5 activities for themselves, 1.2 activities by the child’s father, and 2 activities by other caregivers. The associations between ESAs and children’s development varied by caregiver type. Maternal stimulation was positively associated with children’s total development and personal-social development, early stimulation by other caregivers was positively associated with children’s total development and language development, and paternal stimulation was not associated with any development domain. Other associations included receipt of postnatal care and presence of household toys. While maternal stimulation seemed particularly important, findings regarding non-primary caregivers suggest that ECD programming could also benefit from their inclusion.

## Introduction

The call for investing in optimal early child development has been gaining significant ground at a global scale [1–4]. This is encouraging as about 43% of all under-5 children globally are at risk of not being on track to meet their developmental milestones [1]. In Sub-Saharan Africa, nearly two-thirds of under-5 children are at risk [1].

Both the World Health Organization (WHO) and United Nations Children Fund (UNICEF) provide guidance on responsive feeding and caregiving including age-appropriate early stimulation activities [5,6]. Timing, consistency, as well as intensity of early stimulation activities have been shown to affect different stages of early child development [7–11]. In addition, recent cluster-randomized studies have shown that parenting interventions promoting early stimulation significantly improved children’s cognitive, motor and language development [12–14]. Many studies have reported such findings [11,15–21].

It is important to recognize that children’s developmental outcomes are not only influenced by the mother-child or the father-child relationships but also by interactions occurring within the larger family context [22]. In many countries across East, West, Central and Southern Africa where the average household size maybe relatively large and/or include inter-generational living arrangements [23], it is potentially even more likely that children’s developmental outcomes are influenced by interactions with older siblings, grandparents and other adults living in the same household. According to the Ghana Demographic and Health Survey in 2022, nearly 9 out of 10 married women were employed [24]. Women’s employment, whether seasonal or permanent, could mean that children are often left in the care of other household members during the women’s absence. In some cases, co-wives may also be asked to care for children as 15% of married women reported being in a polygynous union in 2022 [24]. While there is a growing evidence base for the effectiveness of “father-inclusive” parenting interventions [25,26], only a few studies have explored the involvement of other household members in caregiving and early stimulation in the context of low- and middle-income countries [27–30].

In Ghana, the government has prioritized strengthening early childhood development so that more young children meet their full developmental potential [31]. However, Ghana-specific data on responsive caregiving is still limited [32,33] and the role of different caregivers in child development in low and middle income countries (LMICs) needs to be further explored in order to inform programming. This study therefore aims to examine: (1) the type and extent of early stimulation activities being practiced by the mother, father, and other members of the household in Ghana; and (2) the associations between maternal, paternal, and other household member stimulation and children’s development.

## Methods

### Study design

Data for this study come from a larger longitudinal cluster-randomized controlled trial (cRCT) designed to evaluate the impact of the *Integrated Mothers and Babies Course and Early Childhood Development (iMBC/ECD)* program in Northern Ghana and delivered by CRS and Ghana Health Service (GHS) on the mental well-being of mothers with children under two years and the age-appropriate development of their children (ClinicalTrials.gov # NCT03665246; start date 1 Aug 2018, end date 28 Feb 2020). Trial results from this parent study have been published [34].

Ethical approval was received from the Duke University Campus IRB (# 2019−0020) and the Navrongo Health Research Center in Ghana (# NHRCIRB314). All participants signed a written informed consent form, or, if they were illiterate, were read the consent form and provided their fingerprint with a witness signature. The study adhered to the STROBE guidelines.

### Sample description

A total of 32 communities in Northern Ghana were recruited, with half located in the West Mamprusi Municipality (North-East Region; Mampruli is spoken) and half in Nabdam District (Upper East Region; Nabt is spoken). Eligible participants enrolled in women’s groups that received health education administered by CRS and GHS, and half of these groups in each district were randomly assigned to receive the iMBC/ECD program as additional content. A total of 374 women were originally enrolled in the trial. Out of which, 313 were followed up at the first follow-up, and because of missing data, 298 total were included in this analysis [34]. All participants were at least 16 years in age, pregnant at baseline, planned on maintaining residence within the community during the program, and were willing to be followed for up to 24 months.

### Data collection

Data were collected at baseline/pre-intervention while participants were pregnant (August 2018) and immediately post-intervention (July 2019), and ~8 months post-intervention (February 2020). The survey was in English and was translated into Mampruli for West Mamprusi District and Nabt for Nabdam District respondents by trained local research assistants during each data collection interview because these two languages are not commonly written languages. Research assistants received a full week of training prior to data collection that included research ethics, secure tablet use, pre-testing of the surveys, interviewing practice, and appropriate referral procedures. More detail on survey translation and data collection procedures have been previously published [35]. Our analyses used data from the baseline and immediate post-intervention timepoints.

### Primary outcome

#### DMC II.

The Developmental Milestones Checklist-II (DMC-II), developed and tested in low-resource settings, evaluates the development of motor, language, and personal and social skills of children aged 3–24 months [36,37]. The DMC-II has a total development score which is a combination of three subscales scores: DMC motor, DMC language, and DMC personal-social. The total development score as well as the three subscales scores were measured at immediate post-intervention and were standardized by the sample standard deviation.

### Covariates

#### Early stimulation activities.

At immediate post-intervention, mothers were asked whether different type of caregivers (i.e., mothers, fathers, and other members) in the household had engaged in the following early stimulation activities in the past 3 days: (1) reading books or looking at picture books with the child; (2) telling stories to the child; (3) singing songs to or with the child, including lullabies; (4) taking the child outside the home; (5) playing with the child; and (6) naming, counting, or drawing things for or with the child. These questions were sourced from the UNICEF MICS Questionnaire (https://mics.unicef.org/). Maternal stimulation, paternal stimulation, and other household member stimulation were coded as continuous variables where the number of engaged activities is counted by type of caregiver in the household.

Early stimulation during pregnancy was assessed at baseline with four questions: during this pregnancy, how often do you talk softly to him/her and touch belly, sing songs to him/her, tell him/her about his/her family, and dance to music. Each response translated to points: never (0), rarely (1), sometimes (2), and frequently (3), then points were summed for an overall score.

#### Socio-demographic and relational factors.

The following variables were included: number of live children in the household at immediate post-intervention (continuous), total number of people living in the household at immediate post-intervention (continuous), mother’s age at baseline (continuous), child’s age in weeks at immediate post-intervention (continuous), couple’s relationship quality as measured by the communication subscale of the Relationship Quality Index (RQI) [38] at baseline (continuous), mother’s education at baseline (none, primary/post-primary, secondary or higher), moderate to severe household hunger as measured by the Household Hunger Scale (HHS) [39] at immediate post-intervention (yes or no), ownership of children’s toys (homemade toys, household objects as toys, or purchased toys from shops) at immediate post-intervention (yes or no), and self-reported sufficient support from female friends and/or female relatives in the past month at immediate post-intervention (yes or no). Relationship status at baseline is added as descriptive information in Table 1 but is not used in the modeling.

**Table 1:** Study population variables (N = 313), Ghana 2018−19.

	N	Mean [SD] or Percentage
Sociodemographic and relational variables for mother and child		
Mother’s age in years [mean, sd]	313	26.8 [6.6]
Child’s age in weeks [mean, sd]	313	32.0 [10.1]
<13 weeks (0–3 months)	14	4.5
14–26 weeks (3–6 months)	81	25.9
27–39 weeks (6–9 months)	134	42.8
40–52 weeks (9–12 months)	83	26.5
53 + weeks (12 months or more)	1	0.3
Relationship status		
Not living with romantic partner	30	9.6
Living with romantic partner	283	90.4
Couple’s relationship quality (communication) [mean, sd]	298	26.9 [4.5]
Number of children in the household [mean, sd]	313	3.2 [1.8]
Total number of people living in the household [mean, sd]	313	9.1 [3.8]
Mother’s education		
None	144	46.0
Primary/Post-primary	145	46.3
Secondary or higher	24	7.7
Household hunger		
Little to none	219	70.0
Moderate to severe	93	29.7
Missing	1	0.3
Household ownership of toys		
No	96	30.7
Yes	217	69.3
Mother receives support from female friends/relatives		
No	204	65.2
Yes	109	34.8
Early stimulation and development		
Early stimulation activities during pregnancy [mean, sd]	313	1.4 [1.5]
Early stimulation activities with child [mean, sd]		
Maternal stimulation	313	2.5 [1.3]
Paternal stimulation	313	1.2 [1.3]
Stimulation by other household members	313	2.0 [1.5]
DMC-II [mean, sd]		
Total score	313	53.3 [23.2]
Social-Personal subscale score	313	21.6 [10.3]
Motor subscale score	313	25.6 [10.5]
Language subscale score	313	6.1 [4.8]
Health-related factors		
PNC received within 1 week		
No	117	37.4
Yes	196	62.6
Maternal depression (PHQ-9)		
None/minimal	256	81.8
Mild	42	13.4
Moderate to Severe	15	4.8
Child’s health (reported by mother)		
Excellent/very good	220	70.3
Good	69	22.0
Fair/poor	24	7.7
Intervention assignment		
Control	131	41.9
Intervention	182	58.2

Note.

SD = standard deviation; DMC = Developmental Milestones Checklist; PNC = postnatal care; PHQ = Patient Health Questionnaire.

Intervention assignment, couple’s relationship quality, mother’s age, mother’s education, relationship status, polygamy, and stimulation during pregnancy were measured at baseline; rest of the variables were measured at the immediate post-intervention period.

#### Health-related factors.

The following variables were included: self-reported receipt of postnatal care (PNC) within one week of delivery was captured at immediate post-intervention (yes or no). Screening for maternal depression was measured by the PHQ-9 [40,41] at immediate post-intervention (scores 0–4 none/minimal, 5–9 mild, 10 + moderate to severe), and child’s health reported by the mother at immediate post-intervention (excellent/very good, good, fair/poor).

#### Intervention assignment.

Whether a survey respondent’s cluster was randomly assigned to the iMBC/ECD program or to the control group at baseline was included in the models.

### Data analysis

Categorical variables were summarized using counts and percentages, and continuous variables were summarized using means and standard deviations (SD). Linear regression analyses with cluster-adjusted standard errors were performed with the standardized total DMC score, standardized DMC personal-social score, standardized DMC motor score, and standardized DMC language score separately as outcomes. The models included maternal stimulation, paternal stimulation, stimulation by other household members, stimulation during pregnancy, number of live children in the household, total number of people living in the household, mother’s age, child’s age in weeks, couple’s relationship quality, mother’s education, status of moderate to severe household hunger, ownership of children’s toys, support from female friends and/or female relatives, receipt of PNC within 1 week, indication of maternal depression, child’s health (reported by the mother), and intervention assignment. Regression estimates are presented in Table 2. All statistical analyses were conducted in Stata SE 18.0. Given the exploratory nature of this cross-sectional analysis and the correlation among developmental domains, we did not apply formal adjustments for multiple comparisons. Findings should therefore be interpreted as hypothesis-generating rather than confirmatory, with emphasis on effect sizes and consistency across domains.

**Table 2:** Associations between maternal, paternal & other caregiver stimulation and DMC domains, Ghana 2018−19.

	Standardized DMC Total Score	Standardized DMC: Personal & Social Domain	Standardized DMC: Motor Domain	Standardized DMC: Language Domain
***Early stimulation and development***				
Maternal stimulation	**0.07**	**0.12**	0.01	0.05
	[0.002, 0.14]	[0.02, 0.22]	[-0.04, 0.07]	[-0.03, 0.13]
Paternal stimulation	−0.02	−0.03	0.04	−0.08
	[-0.07, 0.04]	[-0.10, 0.03]	[-0.02, 0.10]	[-0.19, 0.03]
Stimulation by other household members	**0.05**	0.02	0.04	**0.09**
	[0.002, 0.09]	[-0.05, 0.08]	[-0.003, 0.09]	[0.03, 0.15]
Stimulation during pregnancy	0.003	−0.01	−0.002	0.04
	[-0.05, 0.05]	[-0.06, 0.04]	[-0.05, 0.05]	[-0.03, 0.12]
***Sociodemographic and relational factors***				
Number of live children in the household	0.03	0.03	0.02	0.04
	[-0.05, 0.10]	[-0.05, 0.10]	[-0.07, 0.10]	[-0.04, 0.12]
Total number of people living in the household	**−0.02**	**−0.02**	**−0.01**	**−0.04**
	[-0.04, -0.01]	[-0.04, -0.0001]	[-0.02, -0.002]	[-0.05, -0.02]
Mother’s age	0.002	0.002	0.002	−0.001
	[-0.02, 0.02]	[-0.01, 0.02]	[-0.02, 0.02]	[-0.02, 0.02]
Child’s age in weeks	**0.07**	**0.06**	**0.08**	**0.05**
	[0.06, 0.08]	[0.04, 0.07]	[0.07, 0.08]	[0.04, 0.06]
Couple’s relationship quality (communication)	0.01	0.001	**0.01**	−0.01
	[-0.01, 0.02]	[-0.01, 0.02]	[0.001, 0.02]	[-0.03, 0.02]
Mother’s education				
None	–	–	–	**–**
Primary/post-primary	0.09	0.14	0.02	0.07
	[-0.07,0.24]	[-0.04,0.32]	[-0.14,0.18]	[-0.11,0.26]
Secondary or higher	0.07	0.14	−0.04	0.13
	[-0.24,0.38]	[-0.27,0.55]	[-0.31,0.22]	[-0.26,0.51]
Moderate to severe household hunger	0.04	0.04	−0.06	**0.26**
	[-0.11, 0.19]	[-0.15, 0.22]	[-0.20, 0.08]	[0.08, 0.43]
Ownership of toys	**0.23**	**0.34**	**0.20**	−0.06
	[0.03, 0.43]	[0.09, 0.59]	[0.002, 0.41]	[-0.31, 0.19]
Support from female friends/relatives	0.13	0.22	0.06	0.04
	[-0.11, 0.38]	[-0.14, 0.57]	[-0.08, 0.19]	[-0.21, 0.29]
***Health-related factors***				
PNC within 1 week	**0.19**	0.14	0.15	**0.26**
	[0.004, 0.37]	[-0.07, 0.36]	[-0.01, 0.31]	[0.05, 0.47]
Depression (measured by PHQ-9)				
None/minimal	–	–	–	–
Mild	−0.19	−0.07	−0.17	**−0.39**
	[-0.43, 0.05]	[-0.33, 0.18]	[-0.39, 0.06]	[-0.71, -0.07]
Moderate to Severe	−0.09	−0.06	0.10	**−0.53**
	[-0.40, 0.22]	[-0.48, 0.37]	[-0.11, 0.32]	[-0.94, -0.12]
Child’s health (reported by mother)				
Excellent/very good	–	–	–	–
Good	0.01	−0.11	0.08	0.12
	[-0.15, 0.18]	[-0.28, 0.06]	[-0.08, 0.24]	[-0.14, 0.38]
Fair/poor	−0.32	−0.30	−0.39	−0.01
	[-0.78, 0.15]	[-0.76, 0.16]	[-0.80, 0.01]	[-0.51, 0.49]
***Intervention assignment***	0.16	0.27	0.07	0.03
	[-0.03, 0.35]	[-0.01, 0.56]	[-0.05, 0.19]	[-0.24, 0.30]
Observations	298	298	298	298

95% confidence intervals in brackets.

DMC = Developmental Milestones Checklist-II; PHQ = Patient Health Questionnaire-9.

Linear regression was used with cluster-adjusted standard errors.

Intervention assignment, couple’s relationship quality, mother’s age, mother’s education, and stimulation during pregnancy were measured at baseline; rest of the variables were measured at the immediate post-intervention period.

## Results

### Descriptive background

**Fig 1:**
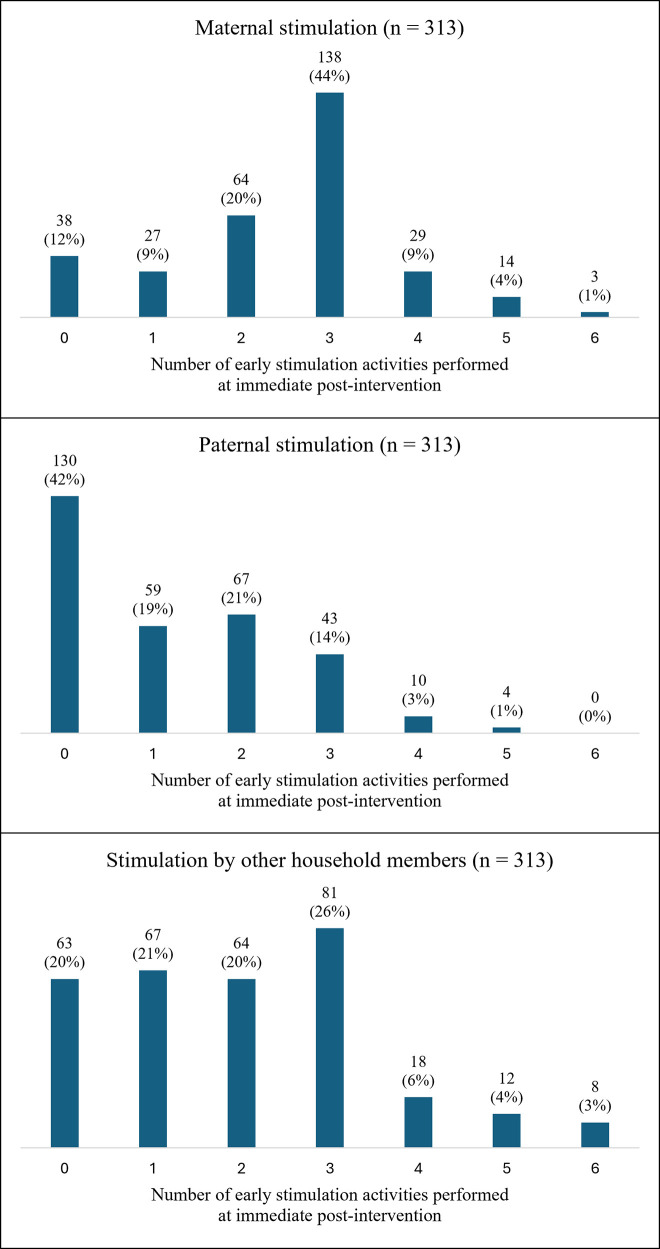
Frequency of maternal, paternal, and other stimulation activities performed in past three days at the immediate post-intervention period (per mothers’ reports), Ghana 2019.

**Fig 2:**
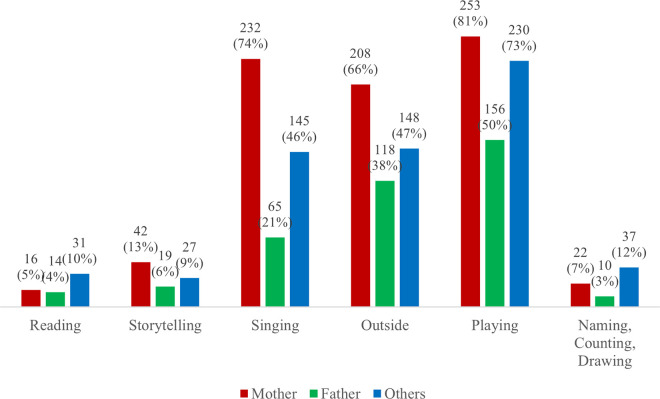
Type of early stimulation activities by caregiver at the immediate post-intervention period (n = 313), Ghana 2019.

Descriptive analysis of the study variables are presented in Table 1, and Figs 1 and 2. Mothers reported an average of 1.4 types of stimulating activities being performed over the course of their pregnancy (in the range of 0–6 activities). At the immediate post-intervention period, mothers reported that an average of 2.5 early stimulation activities were performed by themselves in the past three days (Table 1). Nearly 15% of mothers practiced 4 or more early stimulation activities. There were only 12% of mothers who did not engage in any early stimulation (Fig 1). Mothers reported that an average of 1.2 early stimulation activities were performed by the fathers in the past three days (Table 1). About 42% of the fathers did not engage in any early stimulation and 40% practiced between one and two early paternal stimulation activities (Fig 1). Mothers reported that an average of 2 early stimulation activities were performed by other household members within the past three days (Table 1). About 39% of other household members engaged in 3 or more early stimulation activities and 80% of other household members engaged in at least one early stimulation activity (Fig 1). Across all types of caregivers, singing songs to the child, taking the child outside the home, and playing with the child had the highest frequencies of practice while reading books to the child, telling stories to the child, and naming, counting, or drawing things with the child had the lowest frequencies of practice (Fig 2).

Overall, the mean (SD) total development score measured by the DMC was 53 (23). The mean (SD) personal-social development score measured by the DMC was 22 (10); the mean (SD) motor development score measured by the DMC was 26 (11); and the mean (SD) language development score measured by the DMC was 6 (5), Table 1.

### Multivariable model

The associations between maternal reports of early stimulation activities and children’s development varied by different types of caregivers. Maternal stimulation was positively associated with children’s total development [Estimate: 0.07; 95% CI: 0.002, 0.14] and personal-social development [Estimate: 0.12; 95% CI: 0.02, 0.22] but not associated with motor and language development. Stimulation by other household members was also positively associated with children’s total development [Estimate: 0.05; 95% CI: 0.002, 0.09] and language development [Estimate: 0.09; 95% CI: 0.03, 0.15] but not associated with personal-social development and motor development. However, paternal stimulation was not associated with any development domain. Stimulation by any non-maternal caregiver during pregnancy was also not associated with any development domain (Table 2).

Several socio-demographic, health, and relational factors were associated with different domains of child development. Child’s age in weeks, ownership of toys, couple’s communication quality, experience of moderate to severe household hunger, and receipt of postnatal care within 1 week were positively associated. Larger household size and indications of maternal depression were negatively associated (Table 2).

## Discussion

Early stimulation by mothers and by other members of the household was significantly associated with young children’s overall development. Although the associations were modest in size (0.07 and 0.05 SD, respectively), these estimates represent the marginal association of a single additional stimulation activity for children under one year of age. A similar magnitude of effects was reported from a recent study across 26 sub-Saharan African countries that used identical measures for early stimulation but for children aged 3 and 4 years [42]. In contrast, studies reporting larger effect sizes (around 0.3 SD or higher) typically evaluated structured, multi-component interventions targeting children and their caregivers [43,44]. Maternal stimulation was particularly associated with young children’s personal-social development (0.12 SD), while stimulation by other household members showed the strongest association with young children’s language development (0.09 SD). These findings offer key insight into the important role other household members play in children’s development, especially at earlier stages of development. In many settings, mothers are typically the primary caregivers for young children and parenting programs have mostly focused on the behavior change of mothers and sometimes included participation of their husbands or male partners [25,26,45,46]. Even though discourse around “father-inclusive” parenting interventions has been gaining more traction [25], the co-caregiving role of grandparents, older siblings, and other adult relatives have not been given due consideration in parenting programs.

This is a major gap in programming since mothers, even as the primary caregivers, often have a myriad of other competing household responsibilities [47]. Depending on the context, some mothers may also be running their own small businesses or engaging in family livelihood activities [47]. In Ghana, about 88% of married women were employed and 86% of them were working for payment in cash or in kind other than their housework in 2022 [24], suggesting that employment for married women is very common in these settings. For this reason, other members of the household are likely expected to share in the responsibility of caregiving. Moreover, in cultures and communities where extended family members often live in the same household or within close vicinity of one another, a program that adopts a whole family approach to early child development where non-primary caregivers are also targeted in programming may be more appropriate and reflective of reality and lived experience.

Paternal stimulation was not significantly associated with young children’s development in our models. This is consistent with prior studies reporting minimal effects of paternal stimulation on child development for children under a year old [48,49]. For example, a study in Pakistan found only weak associations between paternal involvement at 3 months and developmental milestones at 12 months [48]. Conversely, other studies found that early paternal engagement during the first year predicts significant improvements in children’s cognitive development [50,51]. Hence, our findings should be interpreted in light of such mixed evidence from the prior literature [48–51]. Nevertheless, the fact that 42% of fathers did not practice any early stimulation activity warrants further attention in this context.

The young age of children in our sample is also why the most frequent types of early stimulation activities are singing songs to or with the child, taking the child outside the home and playing with the child. Reading books or looking at picture books with the child, telling stories to the child and counting or drawing things for or with the child were not highly practiced, perhaps due to low literacy among mothers or because these activities were not considered to be age appropriate by the caregivers. This is also reflected in the low percentage of mothers practicing four or more early stimulation activities. Yet, these activities are encouraged in the UNICEF Care for Child Development Manual as activities beneficial for stimulating children under a year old [5]. The World Health Organization’s updated recommendations on postnatal care also encourage these activities for children 0–3 years [6].

Our study also revealed that a few other covariates had significant associations with young children’s development in Ghana. Ownership of toys at home was significantly associated with children’s development, which is consistent with prior findings that showed a significant association between toy ownership and parental engagement in early stimulation [52]. Receipt of postnatal care within one week of delivery was also significantly associated with children’s development which could suggest a potential relationship between health service use and health education more broadly. The importance of timely and appropriate postnatal care has been promoted not only in the context of increasing the chance of maternal and newborn survival but also for thriving during the critical time period after delivery [6]. Indication of maternal depression was negatively associated with language development. Other studies from different contexts have also found supporting evidence that caregiver mental health plays a vital role in shaping various aspects of child development over time [53–55]. As such, close attention should be given to ensuring caregiver mental health in programming for the dual benefit of promoting maternal health and child development.

Having a large household was associated with lower scores across the development domains. Recent findings from the multi-country Young Lives cohort study also found that having a large household was associated with greater levels of stunting among children [56]. In our sample where experience of moderate to severe hunger is high, time and resources may be spread thin across the household, negatively affecting child development in general. However, a surprising association between hunger and language development in our sample also hints that there could be complex and unexpected pathways that could potentially compensate for lack of resources at the household level. Sensitivity analyses where household hunger was omitted from the models revealed that the associations of early stimulation by different types of caregivers with children’s language development were robust and did not significantly vary in its magnitude and direction (data not shown). Collectively, these findings offer an interesting perspective that just having family members around young children will not necessarily facilitate their development outside of intentionally engaging them in age-appropriate stimulating activities designed to foster optimal development.

There are some limitations in our study. First, early stimulation activities and child development outcomes in the study are measured at the same time point. Hence, we cannot preclude the possibility of bidirectionality but we believe the study findings provide more information than we currently know in the context of Ghana nonetheless. Second, questions about early stimulation activities were from the UNICEF MICS questionnaire and they were originally intended to be about children ages 3 and 4 [33]. However, the study sample included children who were much younger in age. Despite the age gap, we believe that asking caregivers of young children about these activities is still appropriate as similar activities were recommended by international guidance aforementioned [5,6]. Third, early stimulation activities by fathers and other household members were assessed by maternal report which could introduce bias, particularly for activities performed outside of the mother’s direct observation. Fourth, there is potential for selection bias due to attrition from the original sample of 374 participants to the final analytic sample of 298. However, examination of missingness patterns for baseline covariates suggests that any resulting selection bias is likely minimal. In particular, missingness was limited for key covariates (Table in S1 File). Lastly, child development outcomes were measured after a CRS-funded caregiver mental health and early childhood development intervention was implemented [34]. Hence, our findings may not be generalizable to settings where there is an absence of such an ECD-focused intervention despite having controlled for intervention assignment in the analysis. Yet, our findings show that engagement by the whole family can offer significant benefits for children’s development.

## Conclusion

Early stimulation by mothers and other household members was significantly associated with young children’s development. Interventions should not only focus on changing the knowledge, attitudes and practices of mothers and primary caregivers. They should also include specific program components designed for other household members to actively engage in age-appropriate early stimulation and nurturing care to support children’s development.

## Supporting information

S1 File 29Jan2026.docx [Supplemental File 1]. (DOCX)
