# Supplementary material for: Caregivers’ early stimulation behaviors on early child development outcomes in Northern Ghana
Source: PLoS One. 2026 Apr 3;21(4):e0344687. doi: 10.1371/journal.pone.0344687 (PMC13048392; doi:10.1371/journal.pone.0344687)
Supplement: S1 File — (DOCX) [file pone.0344687.s001.docx]

| **Supplemental Table 1. Distribution of baseline covariates by loss to follow-up** | | | | |
| --- | --- | --- | --- | --- |
|  | Non-missing | Missing | Total |  |
|  | (N = 298) | (N = 76) | (N = 374) | p-value |
| **Couple’s relationship quality (communication), Mean (SD)** | 26.9 (4.5) | 26.8 (4.5) | 26.9 (4.5) | 0.889 |
| **Mother’s age, Mean (SD)** | 27.1 (6.6) | 26.5 (7.7) | 27.0 (6.8) | 0.539 |
| **Total number of people living in the household, Mean (SD)** | 8.8 (3.7) | 9.1 (3.9) | 8.8 (3.7) | 0.541 |
| **Stimulation during pregnancy,**  **Mean (SD)** | 1.4 (1.5) | 1.4 (1.5) | 1.4 (1.5) | 0.922 |
| **Household Hunger** |  |  |  | 0.669 |
| Little to none | 219 (73.5%) | 54 (71.1%) | 273 (73.0%) |  |
| Moderate to severe | 79 (26.5%) | 22 (28.9%) | 101 (27.0%) |  |
| **Depression (measured by PHQ-9)** |  |  |  | 0.376 |
| None/minimal | 116 (38.9%) | 23 (30.3%) | 139 (37.2%) |  |
| Mild | 125 (41.9%) | 36 (47.4%) | 161 (43.0%) |  |
| Moderate to Severe | 57 (19.1%) | 17 (22.4%) | 74 (19.8%) |  |
| **Intervention assignment** |  |  |  | 0.111 |
| Control | 128 (43.0%) | 25 (32.9%) | 153 (40.9%) |  |
| Intervention | 170 (57.0%) | 51 (67.1%) | 221 (59.1%) |  |
| **Support from female friends/relatives** |  |  |  | 0.036 |
| No | 192 (64.4%) | 39 (51.3%) | 231 (61.8%) |  |
| Yes | 106 (35.6%) | 37 (48.7%) | 143 (38.2%) |  |
| **Mother’s education** |  |  |  | 0.269 |
| None | 141 (47.3%) | 41 (53.9%) | 182 (48.7%) |  |
| Primary/post-primary | 135 (45.3%) | 27 (35.5%) | 162 (43.3%) |  |
| Secondary or higher | 22 (7.4%) | 8 (10.5%) | 30 (8.0%) |  |
| *All covariates shown were measured at baseline. Covariates that were not measured at baseline were excluded.* | | | | |
